# 14, 15‐EET alleviates neurological impairment through maintaining mitochondrial dynamics equilibrium via AMPK/SIRT1/FoxO1 signal pathways in mice with cerebral ischemia reperfusion

**DOI:** 10.1111/cns.14198

**Published:** 2023-04-05

**Authors:** Jing Tang, Yiang Chen, Jinyuan Li, Shuo Yan, Zenan Wang, Xinyu Deng, Ke Feng, Yanshuo Zhang, Chunrong Chen, Huixia Geng, Yanming Wang, Lai Wang

**Affiliations:** ^1^ The School of Life Sciences Henan University 475000 Henan Province Kaifeng P. R. China

**Keywords:** 14, 15‐EET, cerebral ischemia reperfusion, mitochondrial dynamics, neuronal apoptosis

## Abstract

**Aim:**

To explore whether 14, 15‐EET regulates mitochondrial dynamics to exert neuroprotective effects after cerebral ischemia–reperfusion and its underlying mechanisms.

**Methods:**

The mouse middle cerebral artery occlusion reperfusion model was used to observe brain infarct volume and neuronal apoptosis by TTC staining and Tunel assay, modified neurological severity score to detect neurological impairment, HE staining and Nissl staining to observe neuron damage, western blot and immunofluorescence methods to detect the expression of mitochondrial dynamics‐related proteins, transmission electron microscopy, and Golgi‐Cox staining to detect mitochondrial morphology and neuronal dendritic spines.

**Results:**

14, 15‐EET reduced the neuronal apoptosis and cerebral infarction volume induced by middle cerebral artery occlusion reperfusion (MCAO/R), inhibited the degradation of dendritic spines, maintained the structural integrity of neurons, and alleviated neurological impairment. Cerebral ischemia–reperfusion induces mitochondrial dynamics disorders, upregulates the expression of the mitochondrial division protein Fis 1, and inhibits the expression of mitochondrial fusion proteins MFN1, MFN2, and OPA1, while 14, 15‐EET treatment reverses this process. Mechanistic studies have shown that 14, 15‐EET promotes the phosphorylation of AMPK, upregulates the expression of SIRT1 and phosphorylation of FoxO1, thereby inhibiting mitochondrial division and promoting mitochondrial fusion, preserving mitochondrial dynamics, maintaining neuronal morphological and structural integrity, and alleviating neurological impairment induced by middle cerebral artery occlusion reperfusion. Compound C treatment diminishes the neuroprotective effect of 14, 15‐EET following MCAO/R in mice.

**Conclusion:**

This study elucidates the novel neuroprotective mechanism of 14, 15‐EET, providing a novel approach for the development of drugs based on mitochondrial dynamics.

## Graphical abstract

We demonstrate that 14, 15‐EET alleviates the neurological impairment induced by middle cerebral artery occlusion reperfusion through preserving mitochondrial dynamics as well as maintaining neuronal morphology and dendritic spine via AMPK/SIRT1/p‐FoxO1 signaling pathway.

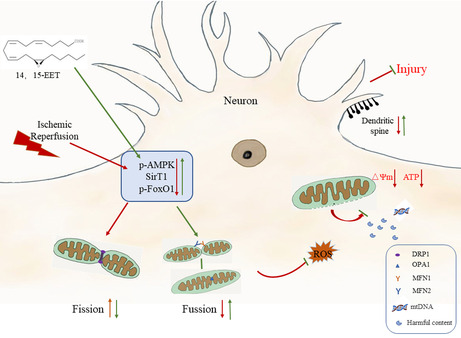

## 1 INTRODUCTION

Stroke is a major cause of disability, and is the second leading cause of death worldwide.^1^ There are approximately 5 million people die each year from stroke globally.^2^, ^3^ Ischemic stroke patients account for 80% of all stroke patients. Removal of blood clots by intravenous recombinant tissue plasminogen activator (t‐PA) or endovascular thrombectomy (EVT) is the most common effective strategy for the treatment of acute ischemic stroke. However, reperfusion injury due to restoring blood flow after vascular unblocking is an important pathologic mechanism that causes neurological impairment in patients.^4^, ^5^ The pathological mechanism of cerebral ischemia–reperfusion is complex, including inflammatory responses, blood–brain barrier destruction, calcium influx, excitotoxicity, oxidative stress, and apoptosis.^6^, ^7^

Mitochondria have various functions as important organelles in cells, including the production of ATP and biosynthetic intermediates, and promote cellular stress responses, such as autophagy and apoptosis. Mitochondrial dynamics, including the fission and fusion of mitochondria, maintain mitochondrial morphological adaptations and are necessary for cell functional stability and survival.^8^ Multiple studies have shown that mitochondrial dynamics disorders are involved in neuronal damage induced by cerebral ischemia–reperfusion.^9^ Mitochondrial dynamics regulate changes in mitochondrial network morphology, which is essential for maintaining mitochondrial homeostasis in cells.^10^ The realization of cellular functional diversity is accompanied by changes in the morphology of mitochondrial networks, such as cell metabolism, pluripotency, division, differentiation, aging, and apoptosis.^11^ After ischemic stroke, insufficient oxygen supply to the brain leads to mitochondrial dysfunction. ATP production is blocked, resulting in cellular energy imbalance. The structurally damaged mitochondria release their contents into the cytoplasm, causing irreversible damage to neurons. Recent studies have found that regulating the fission and fusion of mitochondria to maintain the stability of mitochondrial dynamics is conducive to playing a protective role on neurons.^12^

AMP‐activated protein kinase (AMPK), belongs to the serine/threonine (Ser/Thr) kinase group, and is widely distributed in multiple tissues and organs such as the brain, heart, liver, and kidney.^13^, ^14^, ^15^, ^16^ AMPK detects multiple harmful stimuli and responds to biological responses such as cerebral ischemia, cerebral hemorrhage, and neurodegenerative diseases (NDDs).^17^ Numerous studies have shown that AMPK's regulation of mitochondrial division processes affects mitochondrial dynamics stability, maintains mitochondrial function, and improves brain injury induced after cerebral ischemia–reperfusion.^18^, ^19^ Silent information regulator 1 (SIRT1), as a cellular energy‐sensing molecule, affects the lifespan of an organism by regulating energy metabolism processes.^20^ However, in addition to regulating energy metabolism, SIRT1 is also involved in biological processes such as mitochondrial function, cellular anti‐stress, and protection against neurodegeneration and cardiovascular diseases.^21^, ^22^, ^23^ Forkhead box O (FoxO) is a substrate of SIRT1 and is involved in biological processes such as antioxidant, anti‐apoptosis, mitochondrial quality control, insulin secretion and metabolism.^24^

Epoxyeicosatrienoic acids (EETs) are cytochrome P450 metabolites of arachidonic acid, which play important physiological functions in the body, such as promoting vasodilation, increasing angiogenesis, and reducing pain and inflammation. There are four regioisomeric EETs formed: 5, 6‐EET, 8, 9‐EET, 11, 12‐EET, and 14, 15‐EET. Due to its structure–activity, 14, 15‐EET is readily hydrolyzed to inactive 14, 15‐DHET by soluble epoxide hydrolase.^25^ Our previous studies have shown that 14, 15‐EET reduces neuronal apoptosis caused by cerebral ischemia–reperfusion through regulating mitochondrial biogenesis and inhibiting mitochondrial apoptosis pathways.^26^, ^27^, ^28^ This study intends to investigate the effect of 14, 15‐EET on mitochondrial dynamics of neurons induced by middle cerebral artery occlusion (MCAO) and reperfusion in mice, and to elucidate the neuroprotective mechanism of 14, 15‐EET regulating mitochondrial dynamics through AMPK/SIRT1/FoxO1 signaling pathway to alleviate neurological impairment induced by cerebral ischemia–reperfusion, so as to provide a target for the development of drugs for the treatment of cerebral ischemia–reperfusion.

## 2 MATERIALS AND METHODS

### 2.1 Animals and reagents

Adult male C57BL/6N mice (body weight: 20–23 g, age: 6–7 weeks) were obtained from the Charles River (Beijing, China). Alexa Fluor 488‐ and 532‐labeled fluorescent secondary antibodies, BCA Protein Assay Kit, and other reagents were purchased from Thermo Fisher Scientific Inc (ThermoFisher Scientific, Waltham, MA, USA). The rabbit anti‐AMPK antibody (D5A2), rabbit anti‐SIRT1 antibody (D1D7), rabbit anti‐Phospho‐FoxO1 (Ser256), and rabbit anti‐Phospho‐AMPK (Thr172) were purchased from Cell Signaling Technology (Danvers, MA, USA). The RIPA lysate was purchased from Kangwei Century Biotechnology (Cwbio, Beijing, China). The Tunel assay kits (G1501) were purchased from Wuhan Servicebio Company (Wuhan, China). The MCAO thread plugs were purchased from RWD life science Co., Ltd. (Shenzhen, China). Compound C (866405–64‐3) was purchased from the MCE (Shanghai, China). Other chemical reagents were analytically pure.

### 2.2 Middle cerebral artery occlusion and reperfusion

Male C57BL/6J mice were housed in a specific pathogen‐free (SPF) animal facility with constant temperature, humidity, a 12 h light/dark cycle, and free access to water and food. All procedures were approved by the Ethics Committee of Henan University School of Medicine. The mice were anesthetized with sodium pentobarbital (1%) by intraperitoneal injection. After depilating the neck, the skin was cut with surgical scissors and the muscle layer was separated under a stereomicroscope. The internal carotid artery (ICA), external carotid artery (ECA), and common carotid arteries (CCA) were exposed and ligated with sutures. A filament was inserted into the ECA and fixed with a slip knot. The slip knot on the ICA was untied. The thread plug was turned homeopathically until its top was plugged into the internal carotid artery. In the same direction, the filament was slowly pushed into the upper left and the thread was stopped when a slight resistance was felt. At the same time, the slip knot on the ICA was slightly tied to fix the filament plug, which blocked the middle cerebral artery. Cerebral blood flow (CBF) was measured with a laser Doppler blood perfusion monitor (PeriFlux 5000, Perimed, Sweden). A reduction of 80% in CBF was considered to constitute the success of the MCAO model. The mouse was placed on a constant temperature insulation table (37°C) to maintain body temperature after the operation. After 120 min, the filament was pulled out and the slipknot on the ICA was fastened to prevent bleeding, which was the reperfusion procedure. Before reperfusion, 14, 15‐EET (100 nM) was injected into the tail vein for the EET group. Compound C (2 mg/Kg, body weight) was dissolved in 14, 15‐EET (100 nM) with 50% DMSO in PBS, and administrated in the tail vein at the onset of reperfusion. Meanwhile, the slipknot on the CAA was untied. The MCAO model was completed after suturing the muscle layer and skin layer. The sham group was subjected to the same steps as the MCAO group except for the use of a filament plug and reperfusion.

### 2.3 TTC staining

The mice were anesthetized by an intraperitoneal injection of sodium pentobarbital. The mouse brains were taken out and placed in the mouse brain matrices, frozen at −40°C for 15 min, and cut into slices 2 mm in thickness. The brain slices were quickly placed in 0.5% 2,3,5‐triphenyl tetrazolium chloride (TTC) dye solution and then transferred to a 37°C incubator for 20 min. The slices were fixed with 4% PFA and photographed with a digital camera. The infarct volume was analyzed and calculated with ImageJ software. Calculation formula: infarct volume = (Volume of unlesioned hemisphere − Uninfarct volume of lesioned hemisphere)/Volume of unlesioned hemisphere × 100%.

### 2.4 Tunel assay

The brain was cut into 5 μm slices with paraffin microtome. TUNEL staining was performed with Fluorescein (FITC) Tunel Cell Apoptosis Detection Kit (Servicebio, China) according to manufacturer's instructions. The number of TUNEL‐positive cells and NeuN‐positive neurons were counted in ischemic penumbra of mice with MCAO. Three random sections per brain were picked, and the data were presented as the ratio of TUNEL‐positive neurons (%).

### 2.5 Nissl staining

The mice brain were fixed in 4% (w/v) PFA in PBS, and sliced into sections of 6 μm thickness with freezing microtome (Leica CM1950). For Nissl staining, after dewaxing in xylene and rehydration through graded ethanol, the sections were hydrated in 1% (w/v) toluidine blue at 37°C for 20 min. After rinsing with double‐distilled water, they were dehydrated and mounted with permount. Three slices per brain were used for cell counting. ImageJ software was used to perform quantitative analysis of Nissl bodies' numbers.

### 2.6 Hematoxylin and eosin (HE) staining

Brain sections were prepared in the same manner as Nissl staining. The slices were stained with hematoxylin and eosin. The staining methods were as follows: The tissue sections were immersed in PBS for 3 min, immersed in hematoxylin and stained for 4 min, and rinsed with water for 1 min. The sections were differentiated with 1% hydrochloric acid alcohol for 10 s, rinsed with water for 1 min, made blue color with 1% ammonia water for 30 s, rinsed with water for 30 s, stained with 0.5% eosin solution for 2 min, rinsed with water for 30 s, dehydrated with 95% ethanol twice, and 100% ethanol twice for 30 s, respectively. Drop to drop of neutral resin, seal the tissue slices and photos were taken with the optical microscope.

### 2.7 Modified neurological severity scores (mNSS)

Modified neurological severity score (mNSS) was used to evaluate the neurobehavioral function at 22 h after MCAO. This experiment was carried out by two independent investigators who were blinded to the mice in each group and completed within 15 min. The mNSS consists of motor, sensory, and reflex tests. The mNSS score is graded via a scale of 1–14 points (1–4: mild injury; 5–9: moderate injury; 10–14: severe injury; the scores of 1 and 14 represent normal performance and severe neurological deficit, respectively). In the assessment of neurobehavioral function, the score of 1 represents that the test is uncompleted or there is no corresponding reflection. Therefore, a higher score suggests more severe neurological injury.

### 2.8 Golgi‐Cox staining

The Golgi‐Cox staining was performed to investigate the changes in neuronal morphology and dendritic spines after MCAO reperfusion in this study. All tissues were stained using the FD Rapid Golgi Stain Kit (FD Neuro Technologies) and the staining steps were carried out according to the manufacturer's instructions. The mice were anesthetized with sodium pentobarbital (1%), and killed by decapitation. The brains were removed quickly and placed in a mixture of solution A and solution B (1:1) prepared 24 h in advance. It was not fixed with paraformaldehyde. The impregnation solution was replaced after the first 24 h. Then, the brains were immersed in the mixture for 2 weeks in the dark. Next, the brains were immersed in solution C for a week in the dark, and the solution was replaced after 24 h. All brains were then sectioned into 80‐μm‐thick frozen slices. Next, sections were placed into the mixtures of Solution D, Solution E, and distilled water for 10 min. Sections were then rinsed with distilled water for twice. After that, sections were dehydrated in 50%, 75%, and 95% ethanol and dehydrated for 4 min per concentration gradient. The sections were then dehydrated in absolute ethanol for four times, 4 min each time. Finally, the sections were cleared with xylenes, three times for 4 min each time, and the coverslips were sealed with resin sealing tablets.

### 2.9 Western blot

Western blotting was performed as in previous studies.^4^ Briefly, the protein samples were extracted from the brain tissue of mice using RIPA lysis buffer containing 1% protease inhibitor. The proteins were separated on the appropriate SDS‐PAGE gels and then transferred onto PVDF membranes. After blocking with 5% nonfat milk for 2 h, the membranes were incubated at 4°C overnight with the following antibodies: anti‐AMPK (1:1000), anti‐FoxO1 (1:1000), anti‐SIRT1 (1:1000), anti‐MFN1 (1:1000), anti‐MFN2 (1:1000), anti‐OPA1 (1:2000), anti‐DRP1 (1:1000), and anti‐Fis1 (1:1000). On the second day, the membranes were washed three times with TBST, 10 min each time. The secondary antibody was incubated for 90 min at room temperature, and the membranes were washed three times with TBST for 10 min each time. The ECL supersensitive luminescence solution was used to prepare chemiluminescence.

### 2.10 Immunofluorescence

Brain sections were prepared in the same manner as the TUNEL assay. The absorbent paper was used to wipe the water around the slices and an immunohistochemistry pen to circle the tissue before the primary antibody (rabbit anti‐Fis1 antibody [1:500]; rabbit anti‐OPA1 antibody [1:100]; and rabbit anti‐MFN1/MFN2 antibody [1:100]) was added. After that, the tissues were placed in a wet box overnight at 4°C. The next day, the wet box was taken out and placed at room temperature for 30 min. The slices were washed with PBS three times for 10 min each time. Excessive liquid was wiped off while the tissue pieces were kept moist. Diluted fluorescent secondary antibodies (1:500) were added in the dark, followed by incubation at room temperature for 2 h. The tissues were washed three times with PBS in the dark, 10 min each time, and then mounted with DAPI glycerol. The images were taken with a fluorescence microscope (Olympus, BX61, Tokyo, Japan).

### 2.11 Transmission electron microscope (TEM)

Mice were anesthetized with sodium pentobarbital by intraperitoneal injection, and transcardial perfusion with cold PBS. The brain was separated, and immersed in the 2.5% glutaraldehyde solution within 1 minute. After 12 h of fixation, the brains were quickly cut out into a size of 1 mm^3^, which were washed three times with 1 M PBS, 15 min each time. The samples were added with 1% osmic acid for 1 h, and washed with 1 M PBS Buffer for three times, 15 min each time. The samples were invaded acetone with 30%–50%–70%–80%–90% for 30 min per stage at room temperature, pure acetone for three times, 30 min each time. For anhydrous acetone/embedding medium gradient infiltration, the samples were placed in the embedding plate, polymerized at 45°C for 12 h, then transferred into 60°C for 48 h. The samples were sliced by ultrathin sectioning microtome. The ultrathin section samples were stained with lead citrate for 10 min, washed with decarbonated double‐distilled water three times, stained with uranyl acetate for 30 min, washed three times with double‐distilled water, and observed by transmission electron microscope.

### 2.12 Statistics and data analysis

The data were analyzed with GraphPad Prism 8.0 software. All data were displayed in the form of mean ± standard deviation (mean ± SD). One‐way analysis of variance (one‐way ANOVA) or an independent t‐test was used to compare the difference between groups, and p < 0.05 was considered as a significant difference, p < 0.001 very significant difference.

## 3 RESULTS

### 3.1 14, 15‐EET reduces cerebral infarction volume and neuronal apoptosis induced by cerebral ischemia–reperfusion

**FIGURE 1:**
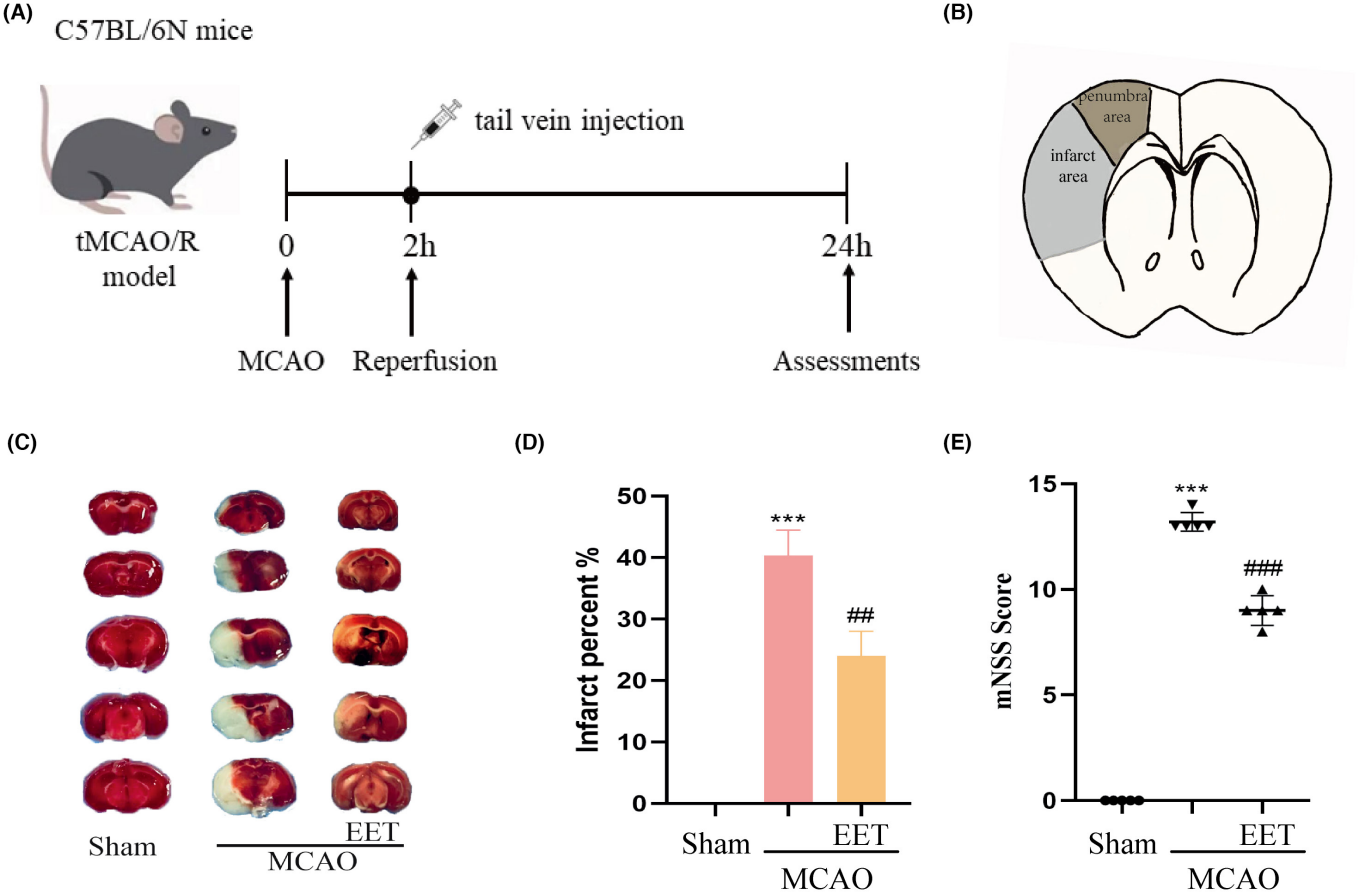
The cerebral infarction area was detected by the TTC staining method after MCAO and reperfusion. (A) A schematic diagram of the experimental schedule. (B) Schematic diagram of cerebral infarct area and penumbra. (C) TTC staining was used in mouse brain tissue sections at 22 h after middle cerebral artery occlusion and reperfusion. The infarct area appears white. (D)Analysis of volume percentage of cerebral infarction (*n* = 3). (E) The mNSS score of the mice at 22 h after middle cerebral artery occlusion and reperfusion (*n* = 5). (***p < 0.001 vs Sham; ^##^p < 0.01, ^###^ *p* < 0.001 vs MCAO).

**FIGURE 2:**
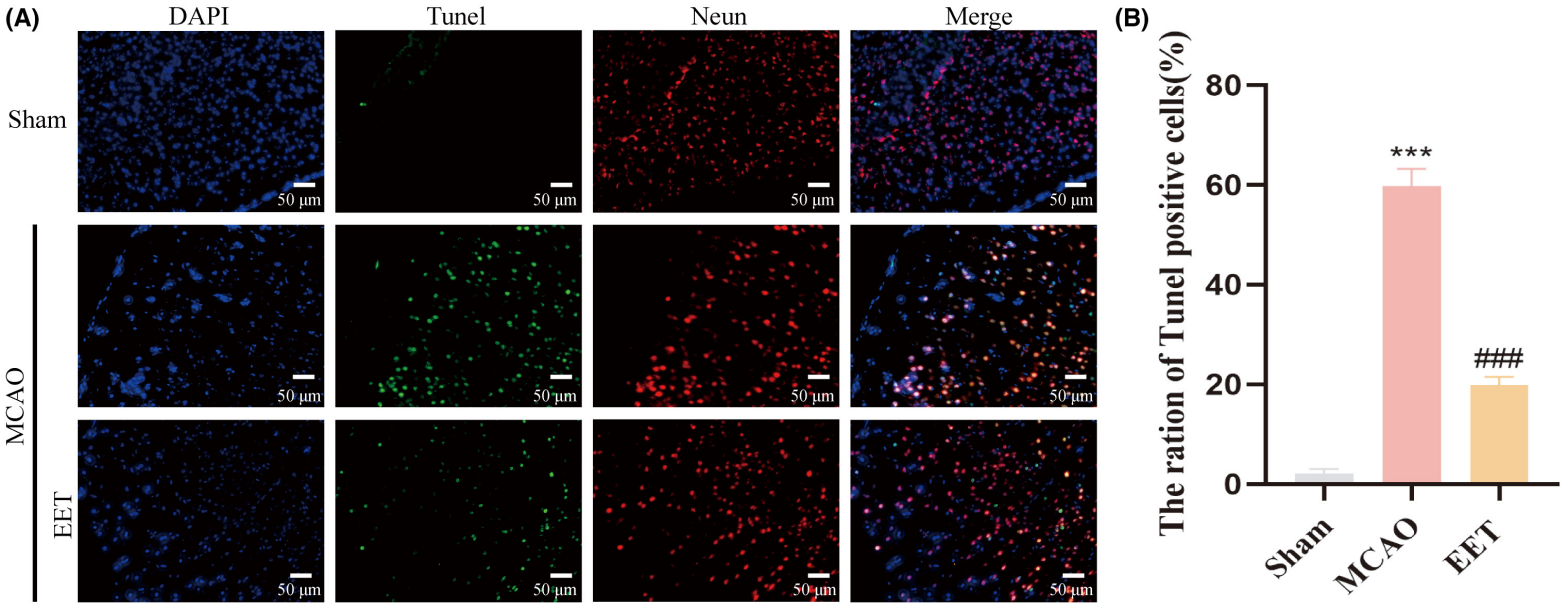
Neuronal apoptosis was detected by Tunel assay after MCAO and reperfusion. (A) Tunel was used to detect neuronal apoptosis at 22 h after middle cerebral artery occlusion and reperfusion in mice. (B) Statistical analysis of neuronal apoptosis in A (20X). (***p < 0.001 vs Sham; ^##^ *p* < 0.01 vs MCAO; *n* = 3).

The experimental schedule is shown in Figure 1A. The middle cerebral artery was occluded for 2 h before reperfusion. At the beginning of reperfusion, 14, 15‐EET (100 nM) was injected into the tail vein, and various indicators were detected 24 h later. The cerebral infarction volume and penumbra after cerebral ischemia–reperfusion are shown in Figure 1B. The results of TTC staining showed that the volume of cerebral infarction increased after cerebral ischemia–reperfusion, while 14, 15‐EET treatment decreased the volume of cerebral infarction (Figure 1C,D), and the difference was significant. The results of the modified neurological severity score (mNSS) showed that the neurological impairment score of mice induced by cerebral ischemia–reperfusion was significantly increased, while the tail vein injection of 14, 15‐EET reduced mNSS, suggesting that 14, 15‐EET treatment alleviated the neurological damage induced by cerebral ischemia–reperfusion (Figure 1E). The results of Tunel staining showed that the apoptotic ratio of neurons in the ischemic penumbra of mice in the MCAO group was significantly increased, while that in the 14, 15‐EET‐treated group was significantly lower than that in the MCAO group (Figure 2).

### 3.2 14, 15‐EET maintains neuronal morphology after cerebral ischemia–reperfusion

**FIGURE 3:**
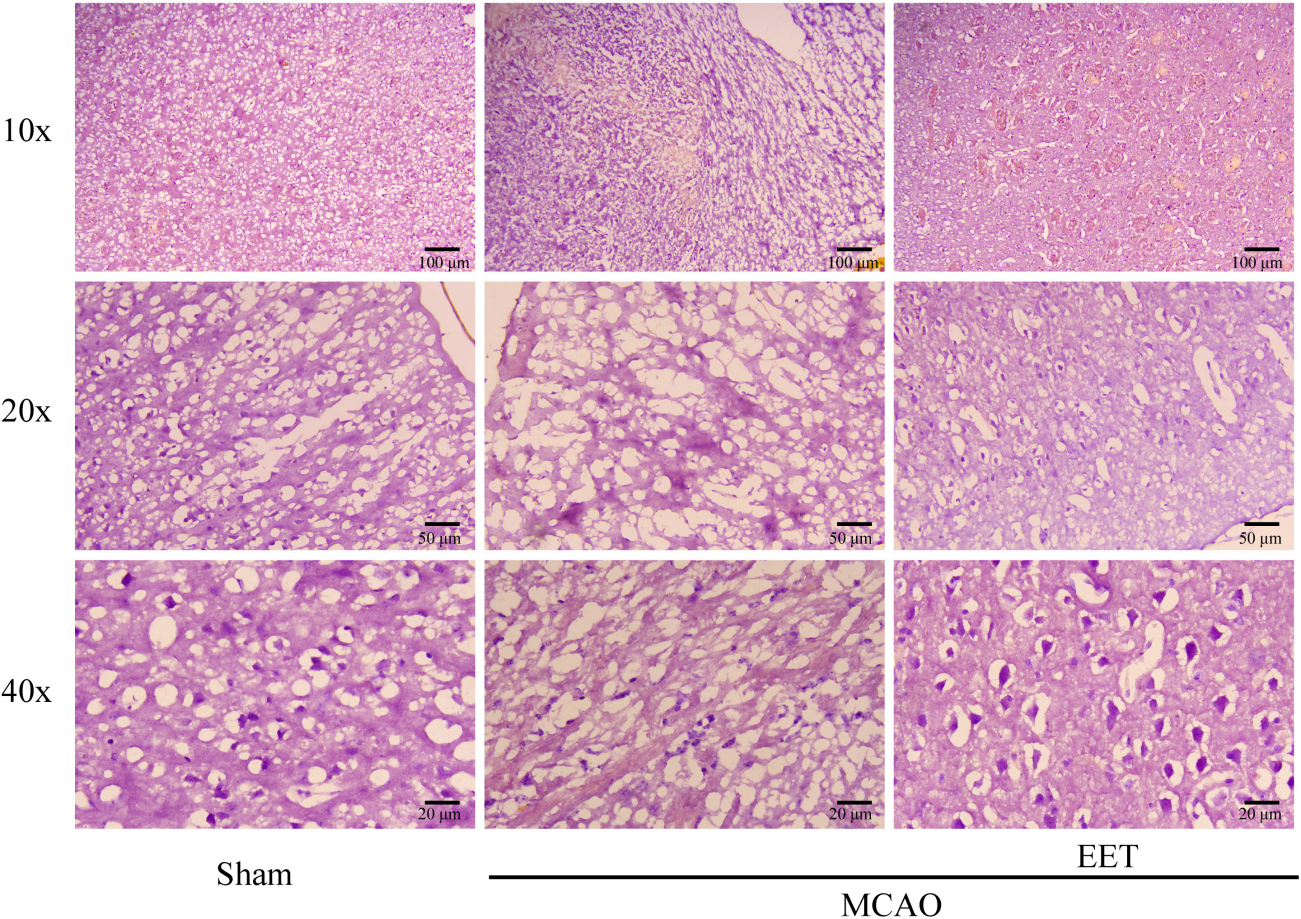
Neuronal structural damages were detected with HE staining.

Hematoxylin–Eosin (HE) staining results showed that the morphological structure of cortical neurons was complete, the nucleus was clearly visible, and the nucleus and cytoplasm were clearly distinguished in the mice in the Sham group. After 22 h of MCAO and reperfusion, the cortical neurons were severely damaged, such as the disappearance of the nuclear structure, shrinkage, deep staining, no complete cell morphology, and a significant increase in the number of damaged neurons. However, after cerebral ischemia–reperfusion in mice treated with 14, 15‐EET, the number of damaged cortical neurons with shrunken and hyperchromatic nuclei was less than that of the MCAO group, while the number of neurons with obvious nucleocytoplasmic structure increased (Figure 3).

**FIGURE 4:**
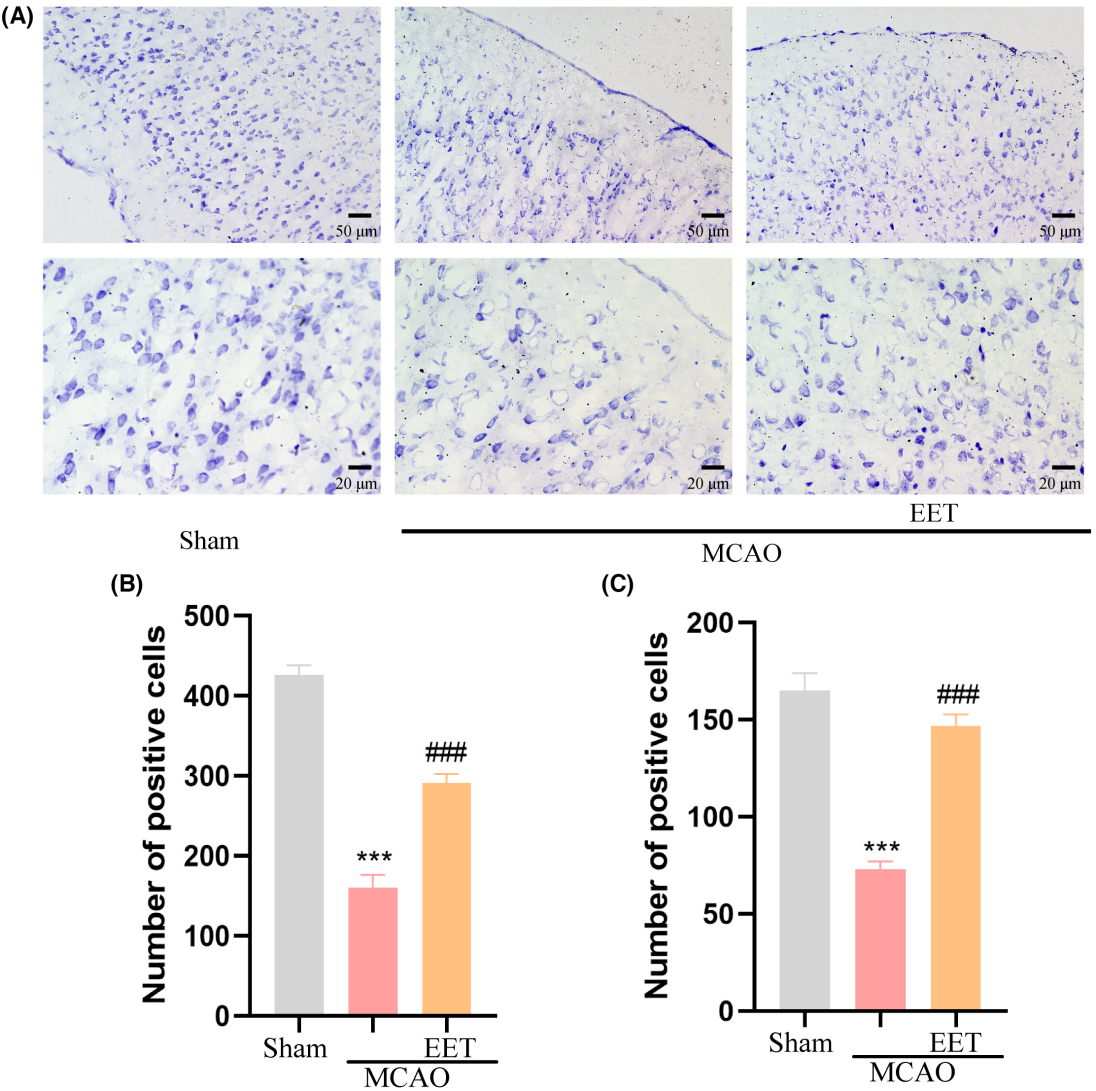
Neuron morphology and structure were detected by Nissl's staining. (A) Nissl's staining was used to observe neuronal morphology at 22 h after middle cerebral artery occlusion and reperfusion in mice. (B, C) Statistical analysis of Nissl staining in A. (***p < 0.001 vs Sham; ^###^ *p* < 0.001 vs MCAO; *n* = 3).

The results of Nissl staining showed that the number of Nissl bodies in the cerebral cortex was large and the shape was complete for the mice in the Sham group, while the number of Nissl bodies reduced in the mice in the MCAO group, and the shape became smaller and irregular. Compared with the MCAO group, the number of Nissl bodies in the cerebral cortex increased and the morphological structure was relatively intact in the 14, 15‐EET treatment group (Figure 4A), suggesting that 14, 15‐EET treatment maintained the structural integrity of neurons after cerebral ischemia–reperfusion.

### 3.3 14, 15‐EET inhibits the expression of mitochondrial fission protein 1 after cerebral ischemia–reperfusion

**FIGURE 5:**
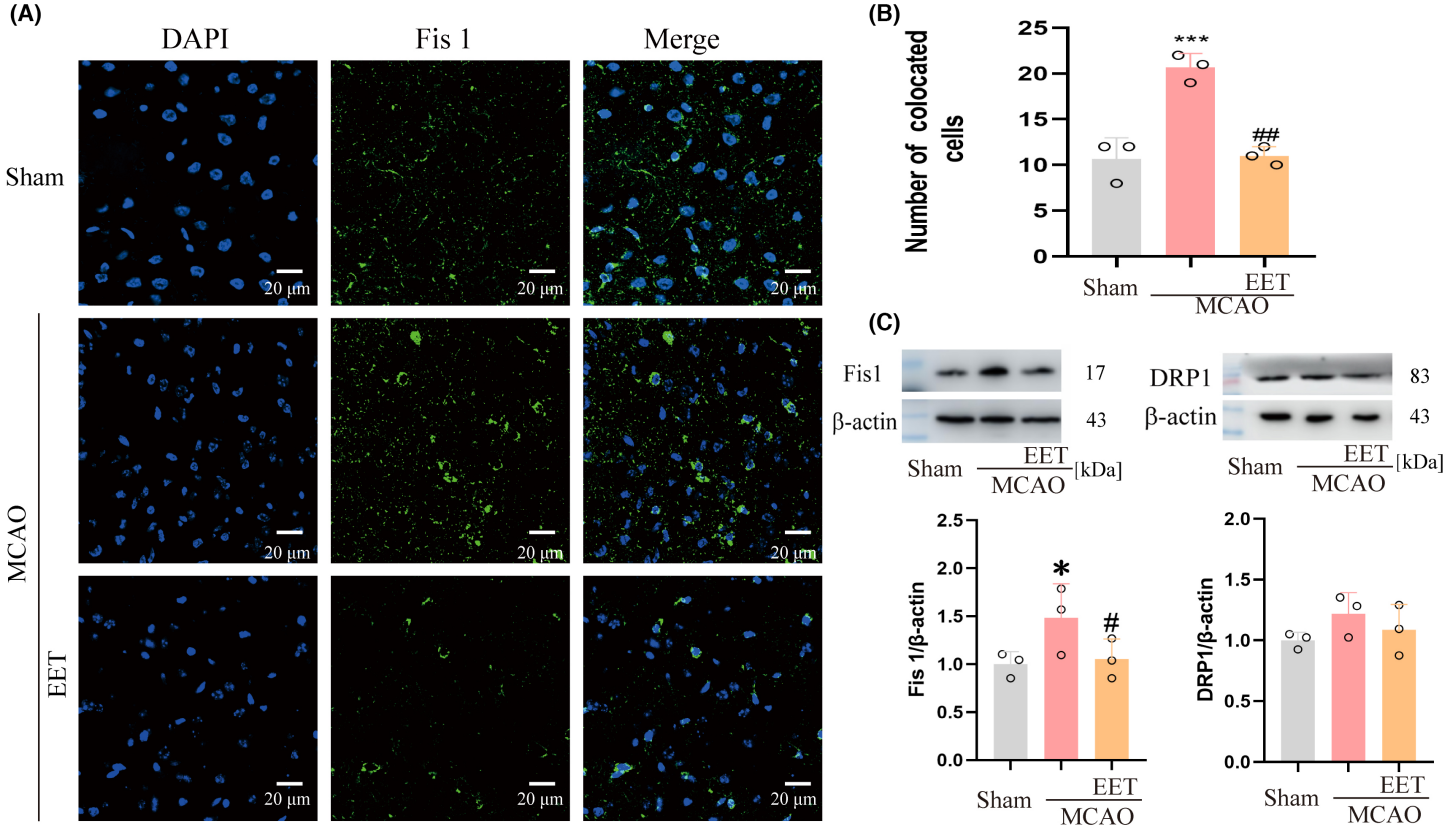
The expression of mitochondrial fission‐related proteins was detected in total brain protein at 22 h after middle cerebral artery occlusion and reperfusion in mice. (A) The immunofluorescence method was used to detect Fis 1 protein expression level (40X). (B) Statistical analysis of immunofluorescence results in A. (C) The expression level of Fis 1 and Drp 1 protein was detected by the western blot method. (*p < 0.05, ***p < 0.001 vs Sham; ^#^ *p* < 0.05, ^##^ *p* < 0.01 vs MCAO; *n* = 3).

The process of mitochondrial fission mainly relies on dynamin‐related protein 1 (Drp1), mitochondrial fission factor (MFF), and mitochondrial fission protein 1 (Fis1) to achieve.^29^ The results with immunofluorescence and western blot showed that the expression of Fis 1 protein increased in mice with the MCAO group, and 14,15‐EET could effectively inhibit this process in the 14, 15‐EET + MCAO group (Figure 5 A,B,C). However, the expression of Drp1 did not receive significant effects on MCAO and 14, 15‐EET (Figure 5C).

### 3.4 14, 15‐EET upregulates the expression of mitochondrial fusion‐related protein after cerebral ischemia–reperfusion

**FIGURE 6:**
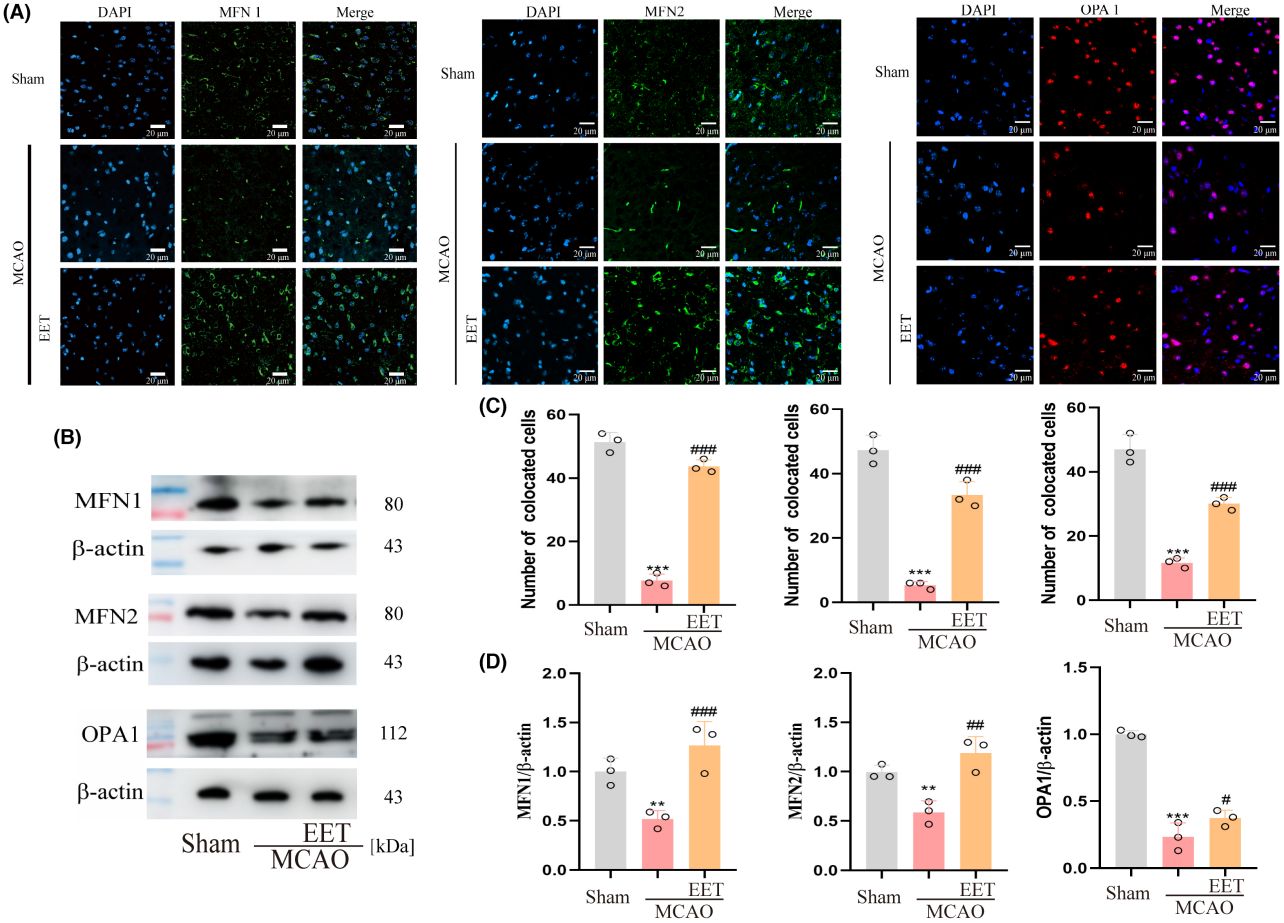
The expression of mitochondrial fusion‐related proteins was detected in total brain protein at 22 h after middle cerebral artery occlusion and reperfusion in mice. (A) The immunofluorescence method was used to detect Mfn1, Mfn2, and OPA1 protein expression level (40X). (B) The expression of Mfn1, Mfn2, and OPA1 protein was detected by the western blot. (C) Statistical analysis of immunofluorescence results in A. (D) Statistical analysis of western blot results in B. (**p < 0.01, ***p < 0.001 vs Sham; ^#^ *p* < 0.5, ^##^ *p* < 0.01, ^###^ *p* < 0.001 vs MCAO; *n* = 3).

Mitofusin 1 (Mfn1), Mfn 2, and optic atrophy 1 (OPA1) are involved in the fusion of mitochondrial outer and inner membranes, respectively, and the coordination of these two processes completes the mitochondrial fusion. Mfn1 and Mfn 2 are responsible for the mitochondrial outer membrane fusion with homodimers or heterodimers.^28^ Immunofluorescence results showed that the expression levels of Mfn1, Mfn2, and OPA1 were downregulated in mouse cerebral cortex after cerebral ischemia–reperfusion, while 14, 15‐EET treatment reversed this protein downregulation (Figure 6A,C). Western Blot results showed that the expression trend of mitochondrial fusion‐related proteins was consistent with the results of immunofluorescence (Figure 6B,D).

### 3.5 14, 15‐EET regulates AMPK/SIRT1/ FoxO1 signal pathway

**FIGURE 7:**
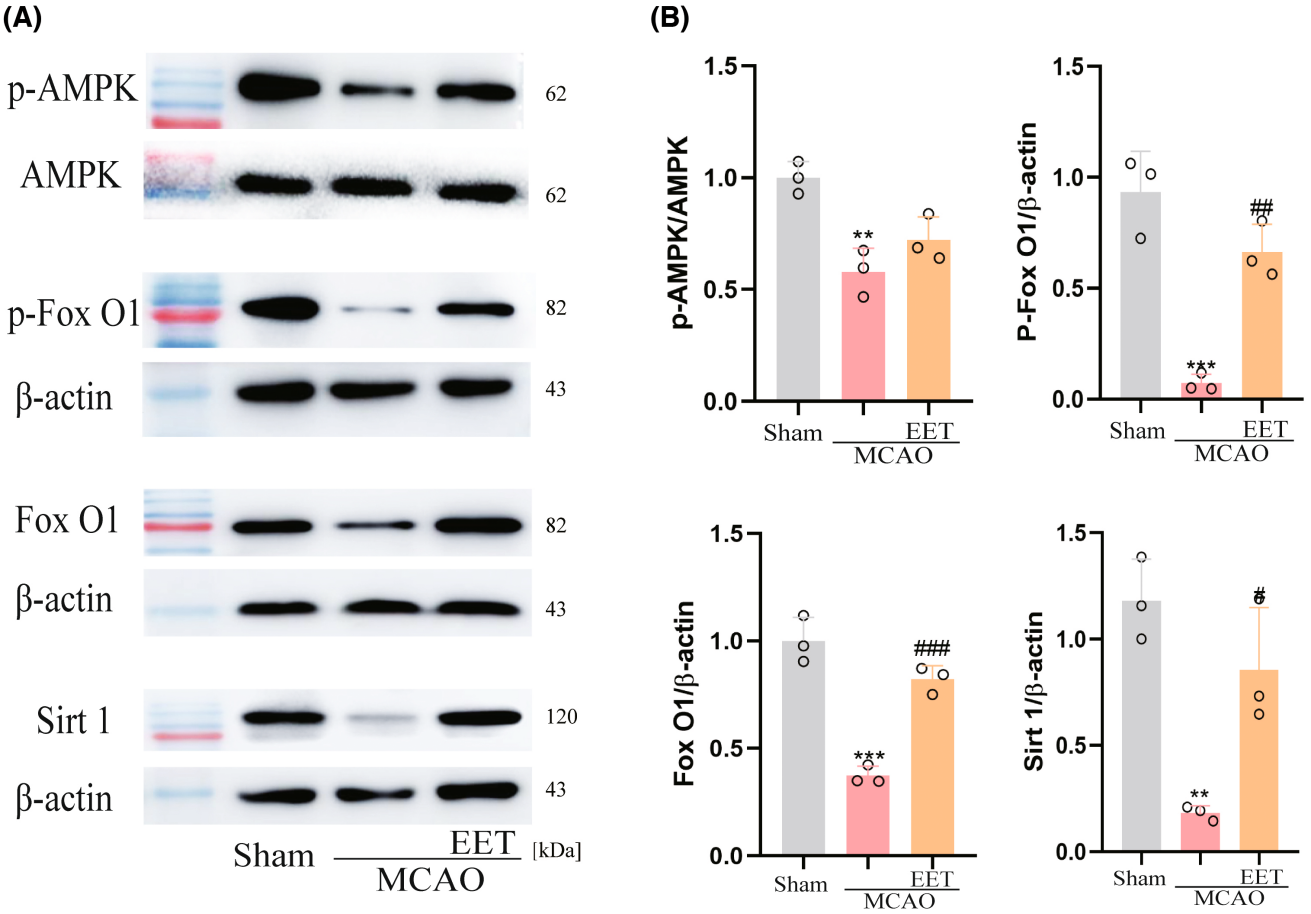
The expression of AMPK/SIRT1/ FoxO1 signal pathway protein was detected in total brain protein at 22 h after middle cerebral artery occlusion and reperfusion in mice. (A)Western blot was used to detect AMPK phosphorylation, SIRT1, FoxO1, and FoxO1 phosphorylation protein levels. (B)Statistical analysis of the western blot results in A. (**p < 0. 01, ***p < 0.001 vs Sham; ^##^ *p* < 0.01, ^###^ *p* < 0.001 vs MCAO; *n* = 3).

After cerebral ischemia–reperfusion, the phosphorylation level of AMPK in the cerebral cortex decreases, resulting in a decrease in the expression of SIRT1, while 14, 15‐EET treatment increases the phosphorylation level of AMPK and upregulates the expression of SIRT1 as well as the phosphorylation of FoxO1 (Figure 7).

### 3.6 14, 15‐EET inhibits mitochondrial fission and promotes mitochondrial fusion to maintain mitochondrial ultrastructure and neuronal morphology

**FIGURE 8:**
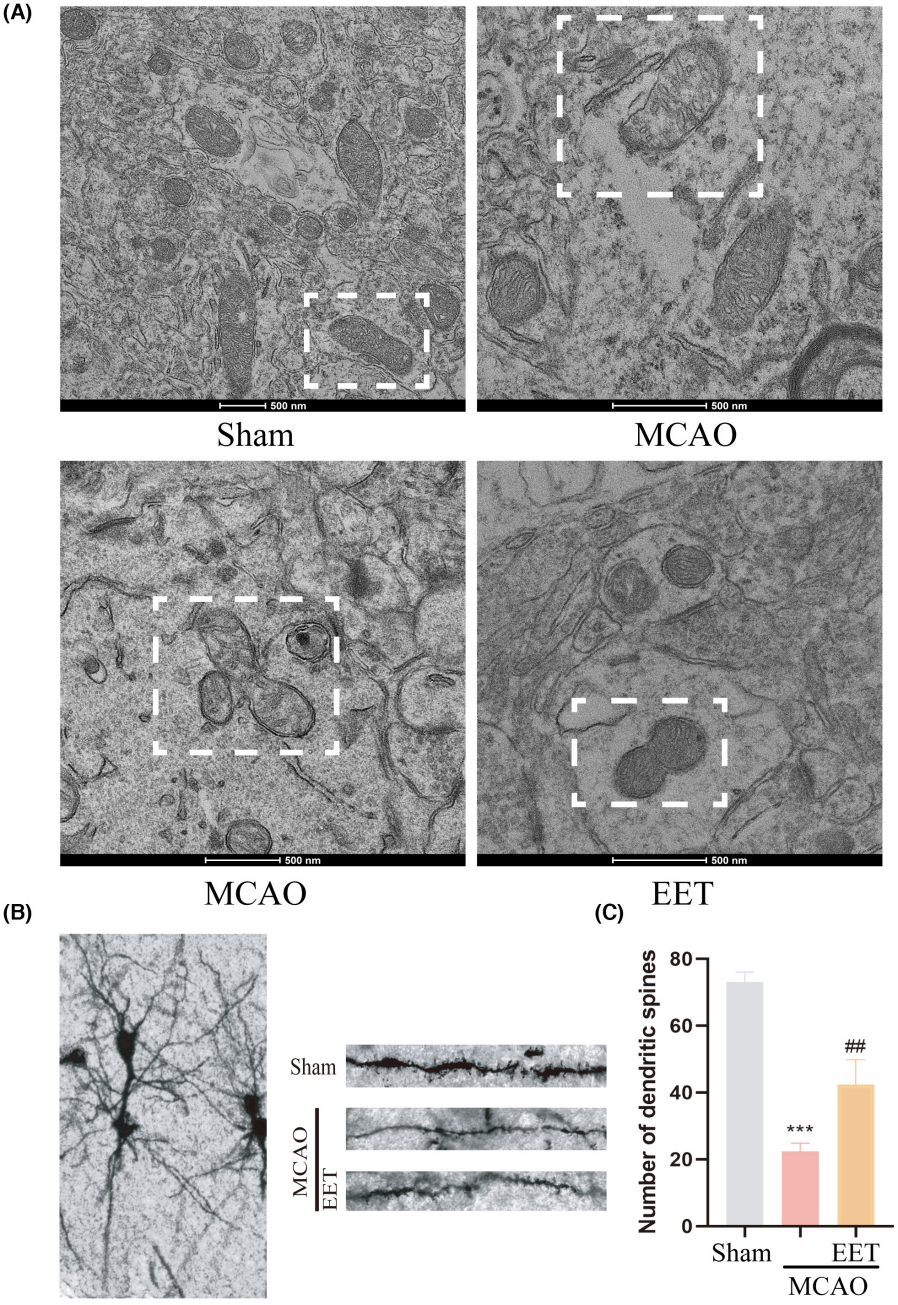
The mitochondrial ultrastructure and neuronal morphology detected in the ischemic penumbra of mice brain after middle cerebral artery occlusion and reperfusion. (A) The mitochondrial ultrastructure and morphology were observed with transmission electron microscopy (TEM). (B) Golgi‐Cox staining was used to detect the neuronal morphology. (C) Statistical analysis of dendritic spine in Golgi‐Cox staining results. (***p < 0.001 vs Sham; ^##^ *p* < 0.01 vs MCAO; *n* = 3).

The results of transmission electron microscopy showed that the mitochondrial membrane structure in the cortical cells of the mice was complete and the cristae were neatly arranged in the Sham group, while mitochondria were swollen, the structure was damaged, the mitochondrial membrane was ruptured, and the contents were released in the MCAO group, as well as irregular stretching of mitochondria and mitochondrial fission both increased (Figure 8A). However, the mitochondrial fission process was inhibited and the fusion process was increased in 14, 15‐EET + MCAO group (Figure 8A). The results of Golgi‐Cox staining showed that the number of dendritic spines in neurons was significantly reduced after cerebral ischemia–reperfusion, while the number and morphology of dendritic spines were maintained with 14, 15‐EET treatment (Figure 8B,C). It can be seen that 14, 15‐EET can maintain neuronal structure and dendritic spine stability by regulating mitochondrial dynamics, and alleviate neurological impairment induced by cerebral ischemia reperfusion.

### 3.7 AMPK inhibitor partially reversed the neuroprotective effect of 14, 15‐EET following MCAO/R in mice

**FIGURE 9:**
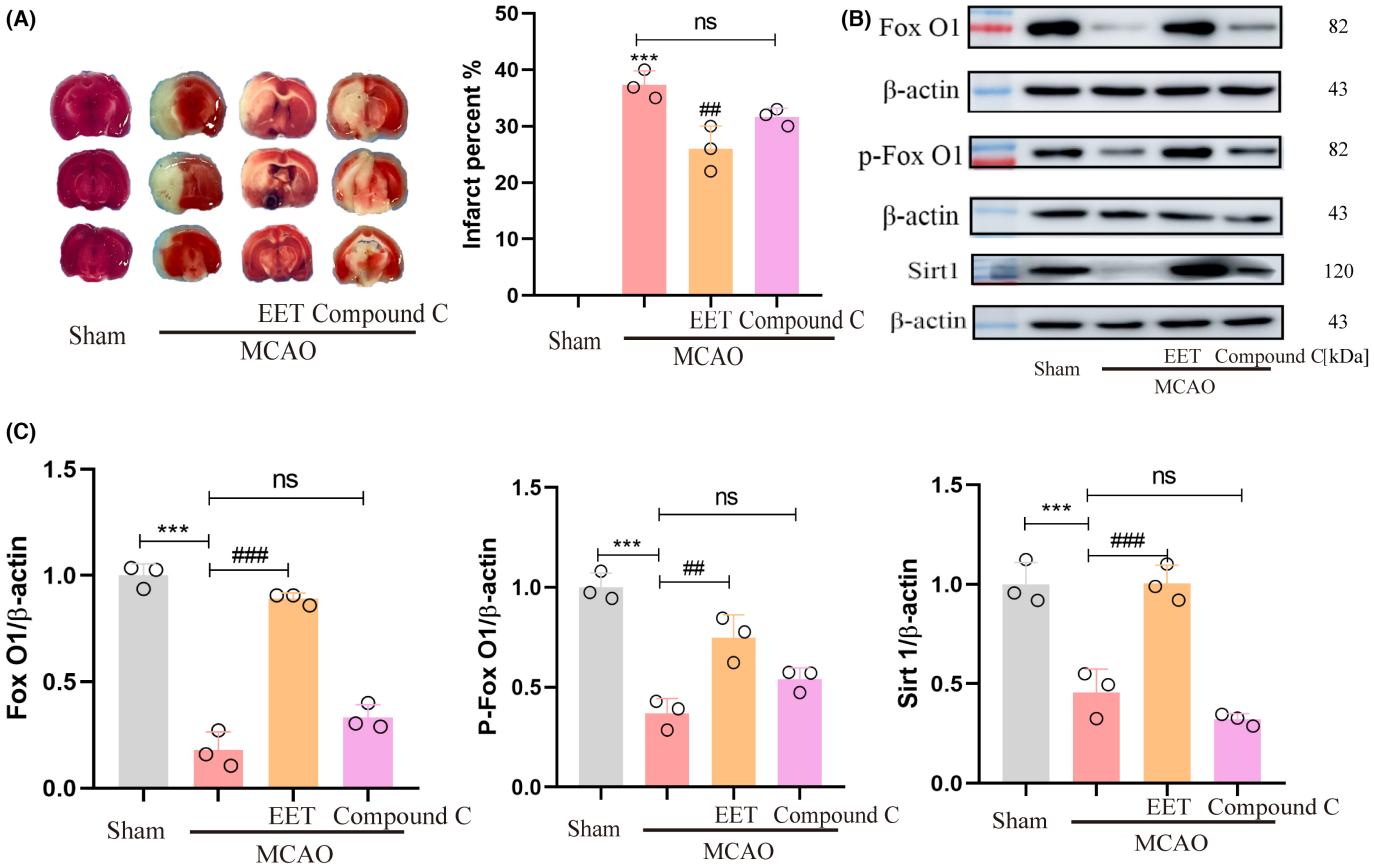
The neuroprotective effect of 14, 15‐EET is partially reversed by compound C treatment A:TTC staining was used in mouse brain tissue sections at 22 h after middle cerebral artery occlusion and reperfusion. The volume percentage of cerebral infarction was analyzed (*n* = 3). (B) The expression of FoxO1, p‐ FoxO1, and Sirt1 protein was detected with western blot method in the brain at 22 h after middle cerebral artery occlusion and reperfusion in mice. (C) Statistical analysis of western blot results in B. (***p < 0.001 vs Sham; ^##^ *p* < 0.01, ^###^ *p* < 0.001 vs MCAO; *n* = 3).

At the onset of reperfusion, 14, 15‐EET + Compound C (2 mg/Kg, body weight) were injected via the tail vein. The results of TTC staining show that the neuroprotective effect of 14, 15‐EET reducing the cerebral infarction volume were partially reversed (Figure 9A). The western blot results showed that the expression of Foxo1, P‐Foxo1, and SIRT1 induced with 14, 15‐EET were inhibited by compound C treatment. The results suggested that the inhibition of AMPK activity eliminated the neuroprotective effect of 14, 15‐EET on cerebral ischemia reperfusion via the AMPK/SIRT1/ FoxO1 signal pathway (Figure 9B,C).

## 4 DISCUSSION

Stroke is one of the most devastating manifestations of cardiovascular and cerebrovascular diseases that causes death and disability worldwide, and ischemic stroke accounts for about 80% of stroke patients. For the treatment of ischemic stroke, there is no effective drug except intravenous recombinant tissue plasminogen activator (tPA) and endovascular thrombectomy (EVT) to remove the blood clots, and restore the blood flow. However, blood reperfusion can easily lead to secondary injury of neurons.^4^, ^5^ Therefore, exploring the protection strategy of neurons after cerebral ischemia–reperfusion is an important strategy to prevent and treat cerebral ischemia–reperfusion injury. 14, 15‐EET is an important intermediate in the process of arachidonic acid metabolism in organisms and has various cytoprotective effects. Our previous study has found that 14, 15‐EET can reduce cerebral ischemia–reperfusion‐induced neuronal apoptosis by promoting mitochondrial biogenesis and inhibiting the mitochondrial apoptosis pathway and parthanatos in neurons.^26^, ^27^, ^28^ This study shows that 14, 15‐EET can reduce neuronal apoptosis and cerebral infarct volume, and alleviate neurological impairment following MCAO, which is consistent with previous findings.

The integrity of mitochondrial quantity and morphological structure maintains mitochondrial homeostasis and ensures the normal function of mitochondria in cells. The homeostatic processes of mitochondrial fission and fusion are known as mitochondrial dynamics.^30^, ^31^ Mitochondrial dysfunction caused by disturbance of mitochondrial dynamics is one of the important pathological mechanisms of cerebral ischemia–reperfusion injury.^29^, ^32^, ^33^ An increase in mitochondrial fission indicates cell apoptosis, and an increase in fusion enhances mitochondrial function and promotes cell viability.^34^ Our study shows that the expression of Fis1 protein is upregulated after cerebral ischemia–reperfusion, which promotes the excessive fission of mitochondria, and the increase of mitochondrial fission will accelerate the process of neuron apoptosis, causing irreversible neural injury.^35^ 14, 15‐EET can effectively inhibit the increase of the Fis1 protein, thereby reducing neuronal apoptosis, and exerting a neuroprotective effect. In addition, mitochondrial fusion plays an important role in mitochondrial homeostasis. Two mitochondria are in close contact. After the mitochondrial outer membrane proteins MFN1/MFN2 are formed in homologous or heterologous complexes, they mediate mitochondrial outer membrane fusion. At this time, the mitochondrial inner membrane potential changes, and the mitochondrial inner membrane fusion is played under the mediation of the OPA1 protein. At this point, the mitochondrial fusion process is completed.^32^ Our study showed that the expression levels of MFN1, MFN2, and OPA1 protein were downregulated after cerebral ischemia–reperfusion, and 14, 15‐EET treatment inhibited the downregulation of these proteins and promoted mitochondrial fusion. The results of transmission electron microscopy also showed that the mitochondrial ultrastructure destruction and the increase of mitochondrial fission were observed in ischemic penumbra of mice in the MCAO reperfusion group, while 14, 15‐EET treatment maintained the stability of mitochondrial structure and promoted mitochondrial fusion. Increased mitochondrial fusion reduces mitochondrial fission, enhances mitochondrial complex III activity, prevents mitochondrial membrane potential loss, and enhances mitochondrial function, thereby inhibiting cell apoptosis.^36^

Adenosine monophosphate‐activated protein kinase (AMPK) is a serine/threonine kinase complex that detects energy status or cellular stress. A growing body of evidence has shown that AMPK specifically regulates mitochondrial biological processes to maintain mitochondrial homeostasis, including mitochondrial biogenesis, mitophagy, etc., and is regarded as a drug target for cardiovascular diseases.^37^, ^38^, ^39^, ^40^ AMPK regulates mitochondrial dynamics, prevents the loss of mitochondrial membrane potential and the opening of mitochondrial permeability transition pore (mPTP), maintains mitochondrial function, and reduces myocardial infarction volume induced by myocardial ischemia–reperfusion.^41^ The results of this study showed that the activity of AMPK was decreased in the brain of mice in the MCAO group, and 14, 15‐EET promoted the upregulation of p‐AMPK, thereby reducing neuronal apoptosis and cerebral infarct volume induced by cerebral ischemia–reperfusion. On the contrary, compound C, an AMPK inhibitor, eliminated the neuroprotective effect of 14, 15‐EET on cerebral ischemia reperfusion by reversing cerebral infarction volume. While AMPK regulates mitochondrial dynamics to maintain mitochondrial homeostasis, it also activates peroxisome proliferator‐activated receptor gamma (PPARγ) coactivator 1α (PGC‐1α), and then upregulates the expression of mitochondrial biogenesis genes and promotes mitochondrial biogenesis.^42^, ^43^, ^44^ Numerous studies have shown that p‐AMPK can upregulate the expression of mitochondrial fusion proteins, including Mfn1, Mfn2, and Opa1, promote mitochondrial fusion, maintain mitochondrial ultrastructure and homeostasis, upregulate ATP synthesis, enhance mitochondrial function, and inhibit cell apoptosis, thereby exerting a cytoprotective effect.^45^, ^46^, ^47^ The results of this study demonstrate that 14, 15‐EET enhances the activity of AMPK and promotes mitochondrial fusion to maintain the stability of mitochondrial dynamics.

Silent information regulator 1 (SIRT1), as the class III of histone deacetylases, plays an important role in physiological and pathological processes such as energy metabolism, apoptosis, and inflammation.^48^ When SIRT1 expression is downregulated, mitochondrial fission is increased, disrupting mitochondrial dynamics, and resulting in irreversible cell damage.^18^, ^49^ This study showed that the expression of SIRT1 in the MCAO group decreased, while 14, 15‐EET treatment promoted the phosphorylation of AMPK and then upregulated the expression of SIRT1, thereby regulating mitochondrial dynamics and reducing neuronal injury induced by cerebral ischemia–reperfusion. Forkhead box O (FoxO) transcription factors are conserved transcription factors that regulate biological processes such as energy metabolism, oxidative stress, cell cycle, apoptosis, and cellular stemness.^50^ Recent studies have shown that FoxO can also regulate mitochondrial function by both direct and indirect means.^51^ Knocking out FoxO1/3 gene expression with shFoxO1/3 in organoids increases mitochondrial fission in cells, suggesting that FoxO 1 can regulate mitochondrial dynamics.^50^ A model study of MCAO reperfusion in rats showed that promoting the phosphorylation of FoxO1 decreased its activity, suppressed neuroinflammation and NLRP3 inflammasome activation and exerted neuroprotective effects.^52^ This study showed that the p‐FoxO1 expression level decreased in the brain of mice in the MCAO group, while 14, 15‐EET pretreatment reversed this effect and further upregulated p‐FoxO1 expression, decreased mitochondrial fission, maintained mitochondrial dynamic stability and structural integrity, inhibited apoptosis, promoted dendritic spine formation, and reduced neurological damage.

**FIGURE 10:**
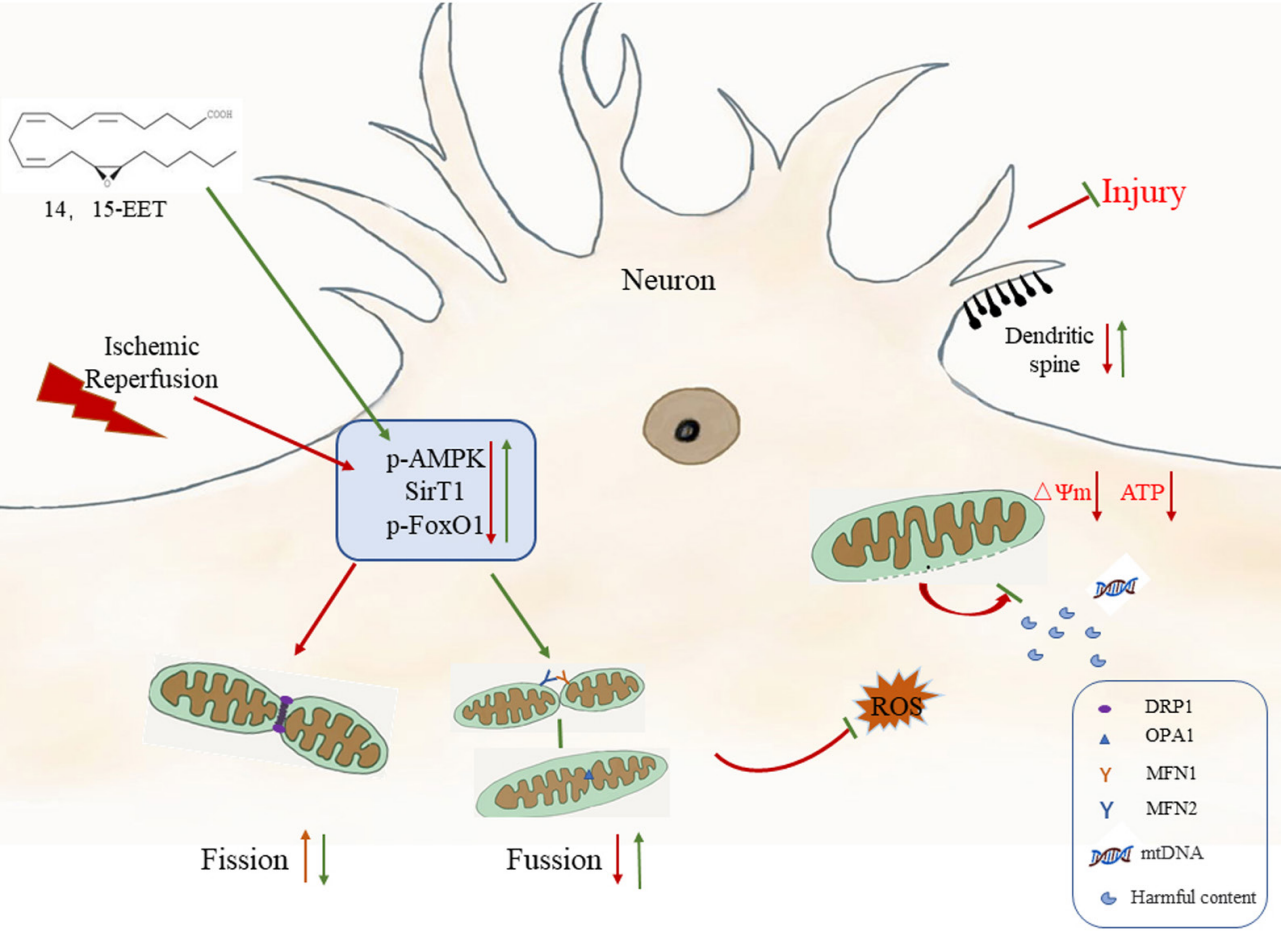
A schematic diagram of 14, 15‐EET inhibition of neuronal apoptosis through maintaining mitochondrial dynamics equilibrium via AMPK/SIRT1/FoxO1 signal pathways.

As intermediates of arachidonic acid metabolism by cytochrome P450 epoxygenases, EETs have various neuroprotective effects.^25^ This study showed that cerebral ischemia–reperfusion increased the expression of mitochondrial fission‐related protein Fis 1 and decreased expression of mitochondrial fusion‐related proteins MFN1, MFN2, and OPA1 in mice brain, resulting in mitochondrial dynamics disorder and structural damage, aggravating neuronal apoptosis and neurological impairment. 14, 15‐EET treatment upregulates the expression of mitochondrial fusion proteins by regulating the AMPK/SIRT1/p‐FoxO1 signaling pathway, inhibits mitochondrial fission, maintains mitochondrial homeostasis, reduces neuronal apoptosis, promotes dendritic spine formation, and alleviates neurological impairment (Figure 10). The results of this study demonstrate that 14, 15‐EET regulates mitochondrial dynamic homeostasis and inhibits neuronal apoptosis induced by cerebral ischemia‐reperfusion, which not only improves the neuroprotective theory of EETs, but also provides a new target for the development of drugs for the treatment of cerebral ischemia‐reperfusion.

## AUTHOR CONTRIBUTIONS

Jing Tang, Yiang Chen, Jinyuan Li, Shuo Yan, Zenan Wang, Xinyu Deng, and Ke Feng performed experiments. Jing Tang, Jinyuan Li, Yanshuo Zhang, and Chunrong Chen analyzed the data, Huixia Geng and Lai Wang designed this research, and contributed to writing the article. Yanming Wang and Lai Wang edited the article. Huixia Geng and Lai Wang provided financial support. All authors have read and agreed to published version of the article.

## FUNDING INFORMATION

This research was funded by National Natural Science Foundation of China (NSFC) grants (81871856), Henan Province Science and Technology Research and Development (222102310434, 232102310495), Innovation and entrepreneurship training program for college students of Henan University (20221014003, 20221014008), and Research Student Excellence Program of Henan University.

## CONFLICT OF INTEREST STATEMENT

The authors declare no conflict of interest.

## Supporting information


Data S1.

## Data Availability

The original contributions presented in this study are included in the article materials; further inquiries can be directed to the corresponding authors.
