# Supplementary material for: 14, 15‐EET alleviates neurological impairment through maintaining mitochondrial dynamics equilibrium via AMPK/SIRT1/FoxO1 signal pathways in mice with cerebral ischemia reperfusion
Source: CNS Neurosci Ther. 2023 Apr 5;29(9):2583–96. doi: 10.1111/cns.14198 (PMC10401172; doi:10.1111/cns.14198)

Full unedited gel/blot for Figure 5(FIS 1 、 DRP1)

FIS 1 17kDa←

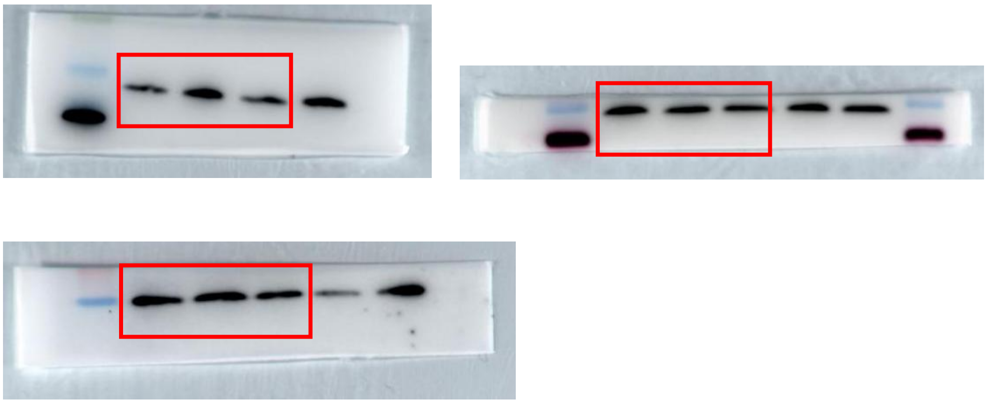

Actin:(43 kDa)

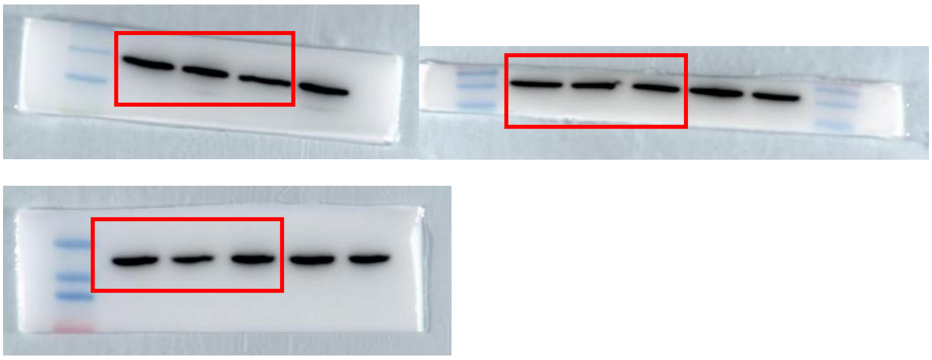

DRP1 83kDa

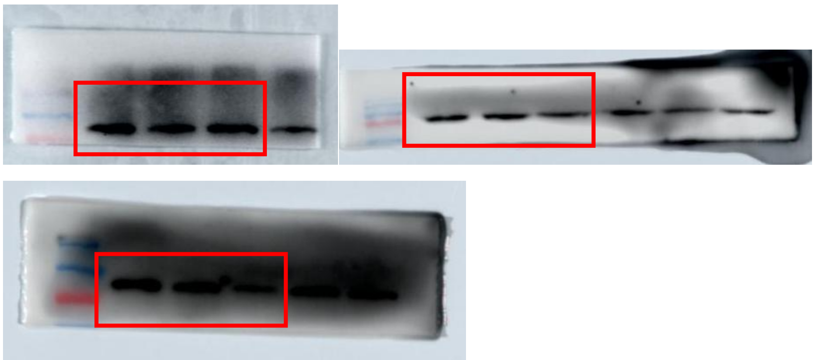

Actin:(43 kDa)

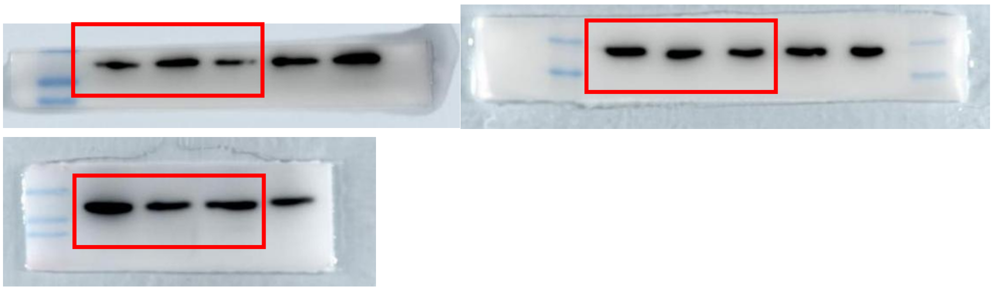

Full unedited gel/blot for Figure 6(MFN1 MFN2 OPA1)

MFN1 80kDa

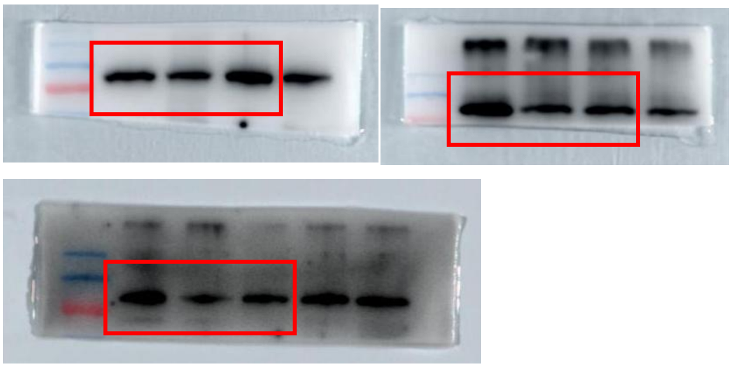

Actin 43 kDa

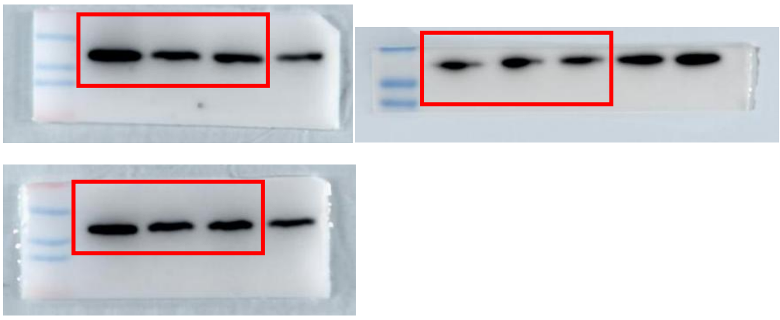

MFN2 80kDa

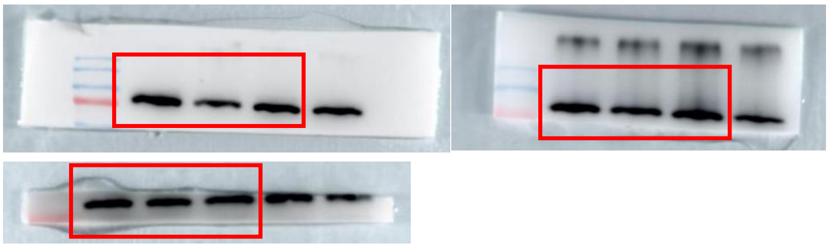

Actin 43 kDa

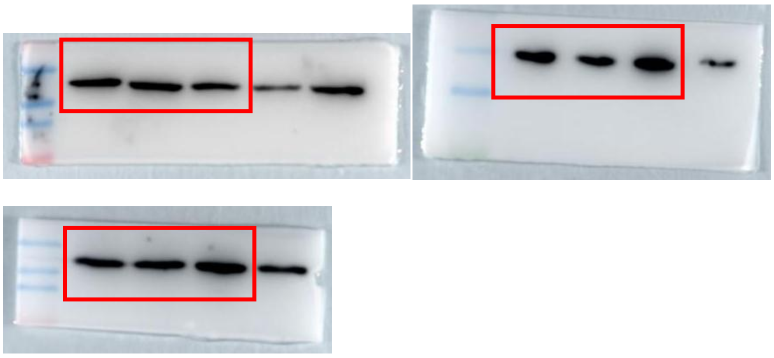

OPA1（112 kDa）

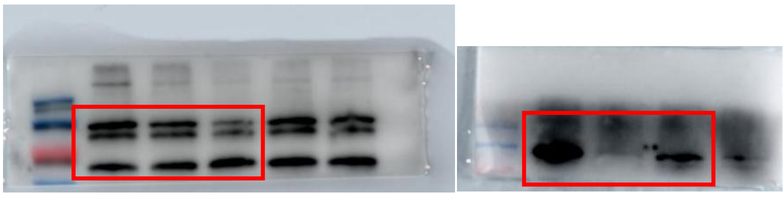

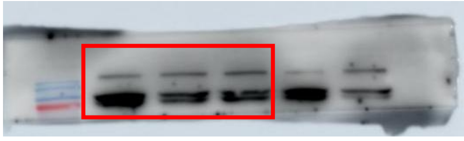

Actin 43 kDa

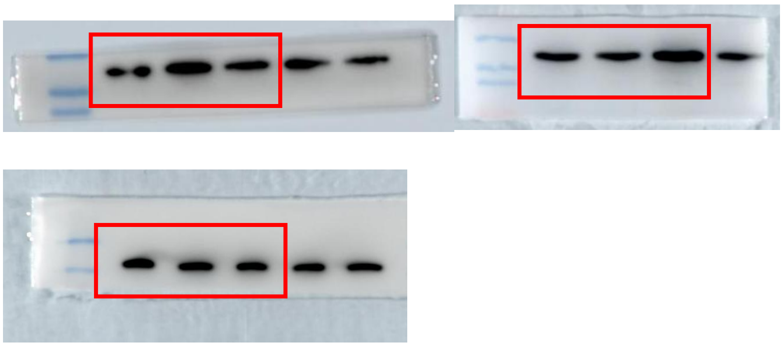

Full unedited gel/blot for Figure 7(p-AMPK 、P-Fox O1、Fox O1、AMPK-a、Sirt 1)

p-AMPK 62kDa

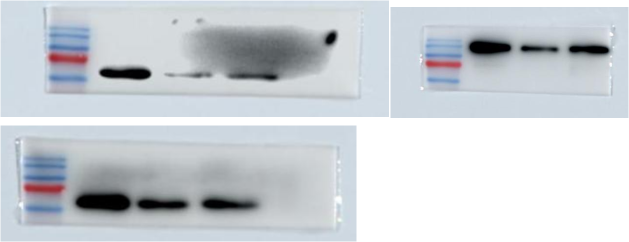

AMPK-a 62kDa

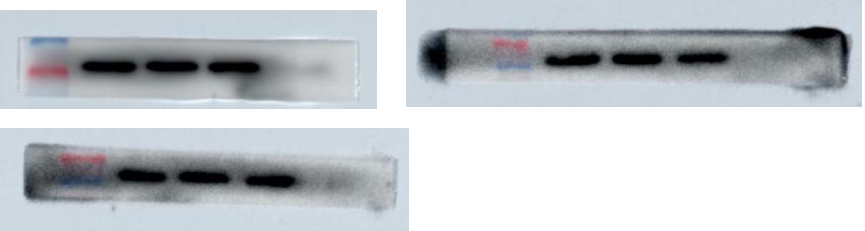

P-Fox O1 82kDa

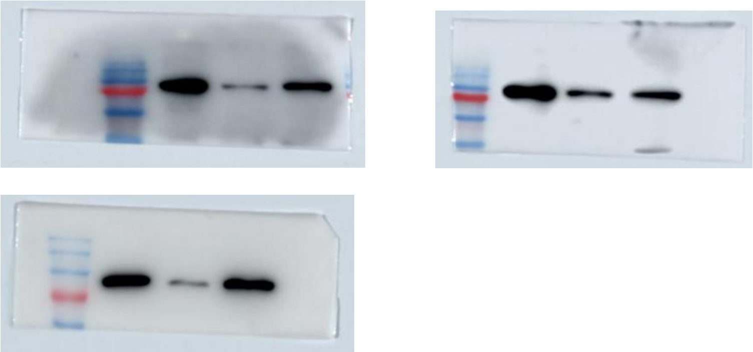

Actin 43 kDa

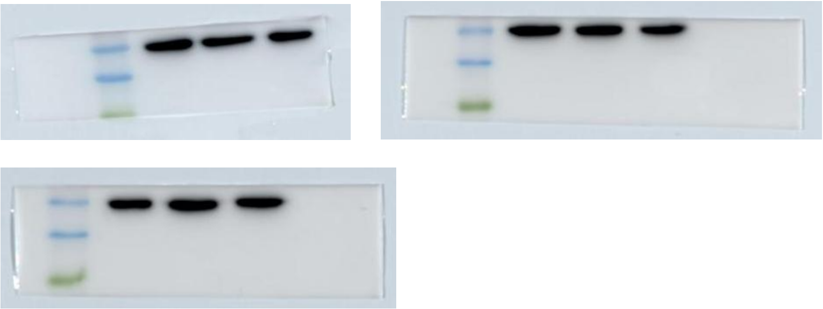

Fox O1 82 kDa

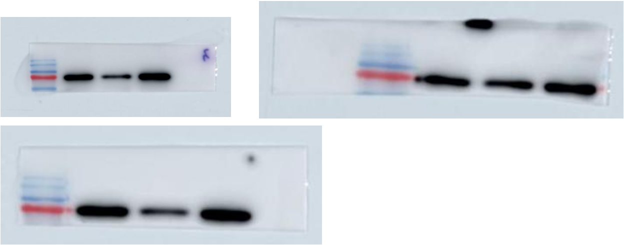

Actin 43 kDa

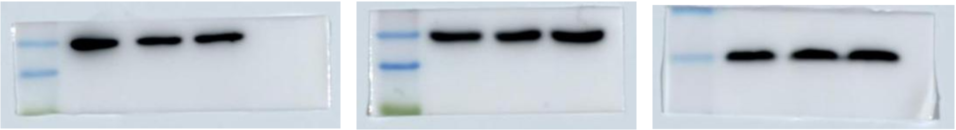

Sirt 1 120kDa

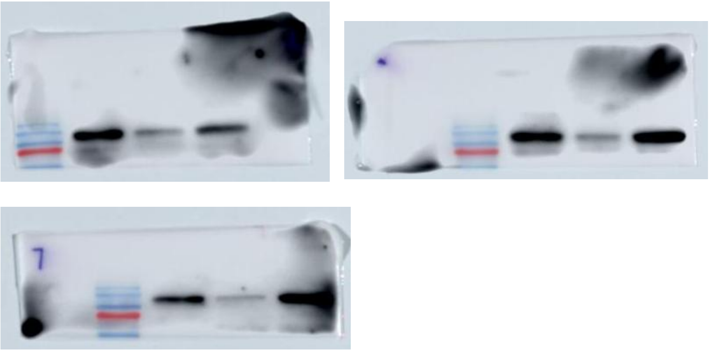

Actin 43 kDa

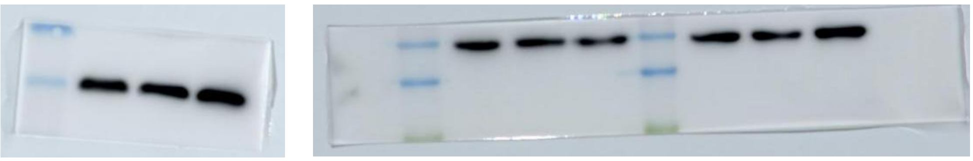

Full unedited gel/blot for Figure 9（FoxO1、p-Fox O1）

Fox O1 82 kDa

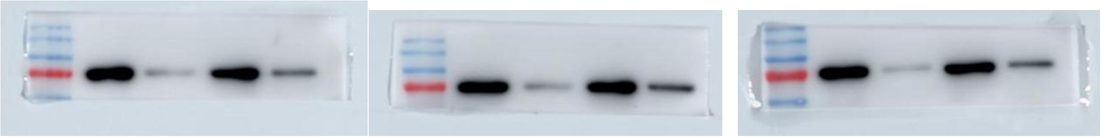

Actin 43 kDa

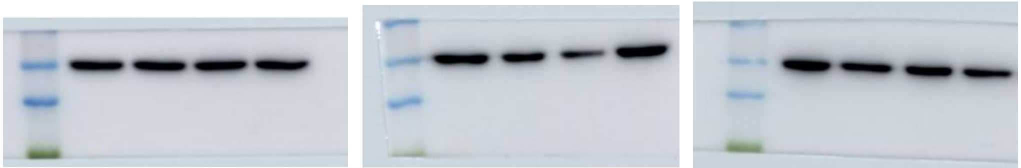

p-Fox O1 82kDa

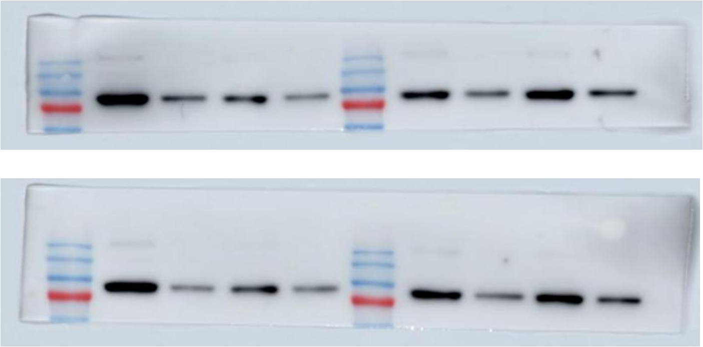

Actin 43 kDa

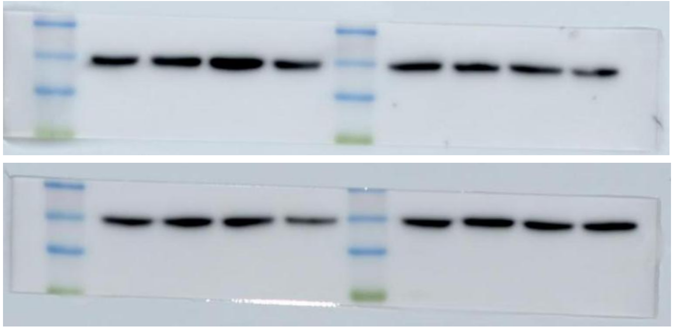

Sirt1 120 kDa

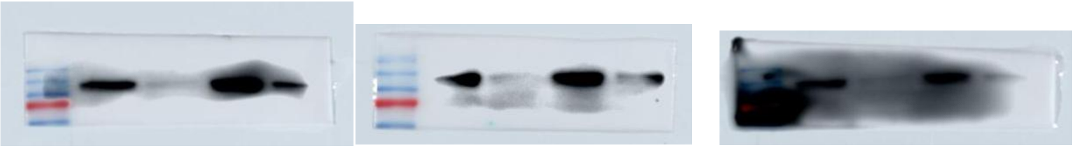

Actin 43 kDa

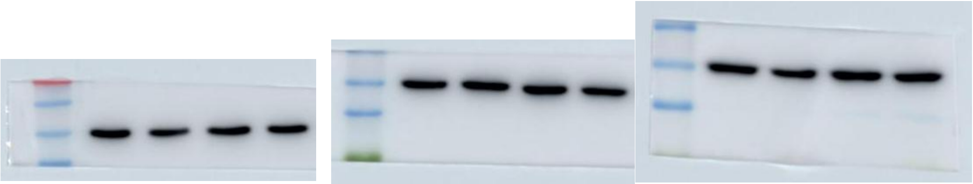

Supplement: Supplementary file 1 — Data S1. [file CNS-29-2583-s001.pdf]
